# Six Weeks of Baker’s Yeast β-Glucan Supplementation Reveals Unique Immune Maturation mRNA Signature: Implications for Immunity?

**DOI:** 10.3390/ijms27020588

**Published:** 2026-01-06

**Authors:** Brian K. McFarlin, Anyla L. Paschall, David G. Cooper, Caleb A. Class, Meredith A. McFarlin

**Affiliations:** 1 Bioanalysis Center, University of North Texas, Denton, TX 76205, USA; 2 Applied Physiology Laboratory, University of North Texas, Denton, TX 76205, USA; 3 College of Pharmacy & Health Sciences, Butler University, Indianapolis, IN 46208, USA

**Keywords:** immune modulation, nutritional immunology, functional genomics, multiplex molecular analysis

## Abstract

Baker’s yeast beta-glucan (BYBG) supplementation improves various aspects of immune system function, readiness, and response. The purpose of this study was to determine if the expression of immune maturation mRNA was also changed over the course of 6 weeks of BYBG supplementation at rest. In this exploratory study, a small group of participants (*N* = 20) were randomized into two groups: BYBG (weeks 0–2 = 50 mg/d; 2–4 = 125 mg/d; and 4–6 = 250 mg/d) or placebo. Blood samples were collected at 0, 2, 4, and 6 weeks and analyzed for the expression of 785 mRNA (NanoString nCounter platform and Nanotube software; R v3.3.2). A total of 42 mRNAs in 21 annotated pathways (antigen presentation, apoptosis, B cell memory, cell cycle, chemokine signaling, cytotoxicity, DAP12 signaling, hypoxia response, IL-1 signaling, IL-10 signaling, MAPK signaling, myeloid immune response, NF-kB signaling, NK activity, Notch Signaling, PD1 signaling, Senescence/Quiescence, T cell checkpoint signaling, TCR signaling, TLR signaling, and TNF signaling), were significantly affected by BYBG at various time points. It is reasonable to speculate that the observed mRNA and associated pathways may underlie previously reported improvements in immune function with BYBG.

## 1. Introduction

Oral supplementation with Baker’s yeast beta-glucan (BYBG) results in immune modulation and innate immune training [1,2,3,4,5,6,7,8,9,10]. While beta-glucans can be found in a variety of natural sources, BYBG has a unique, branched structure that confers its effectiveness on both immune system readiness and response [11,12,13,14,15,16,17,18]. Previous studies have measured the effectiveness of BYBG by provoking the system with an exercise stress model that is designed to mimic the immune response to an antigen [8,9,13,16,18] or a similar immune system challenge [11]. While this experimental approach is effective, it is not easily translated to free-living populations. It would be ideal to identify novel biomarkers that correlate with traditional immune functional measures but are more sensitive to small changes in immune outcomes and better suited for field-based measures. Our lab and others have proposed that, while not a perfect substitute, the measurement of multiple mRNA may represent a suitable alternative to traditional measures due to a high degree of precision and low measurement error [19]. Molecular mRNA biomarkers have long been utilized in pharmaceutical research due to their heightened sensitivity compared to traditional laboratory assays [20,21,22]. Another advantage of using multiplex mRNA detection is that the sensitivity of the assay generally requires fewer analysis replicates to identify significance, which is an advantage in an exploratory trial such as the present study. Once a potential candidate mRNA is identified in a preliminary study, then this can serve as the basis for a larger study with larger enrollment seeking to link mRNA and functional biomarkers of immune system readiness and response.

Our laboratory has conducted several studies using multiplex mRNA expression profiling to assess the biological effects of various botanical and natural compounds on human health [19,23,24,25,26,27,28]. The NanoString analysis approach is well documented and used in scientific literature and is well suited for both small- and large-scale studies. Specifically, in the context of BYBG, we have previously reported the change in expression of several innate immune-related mRNA that are modulated both at rest and in response to exercise-induced stress models [12,14,26]. An existing gap in the BYBG literature, addressed by the present study, is the identification of immune cell maturation mRNA that may be altered by BYBG supplementation, absent of a physical stressor. The purpose of the present exploratory study was to extend our previous findings and identify additional novel immune cell maturation mRNAs that may be changed by 6 weeks of BYBG supplementation. The larger aim of our BYBG research is to refine experimental models such that we can conduct well-designed, large-scale field-based interventions to evaluate the effectiveness of BYBG in free-living populations and/or immune-compromised participants.

## 2. Results and Discussion

### 2.1. Overview of Findings

As noted above, the most common means to assess the impact of BYBG on immune response is to use either a physical stressor or a similar infectious challenge [8,9,13,16,18]. While this approach is well suited to the controlled confines of a laboratory environment, it does not translate well to field-based experimental models. We recently demonstrated that at rest, it was possible to detect changes in the expression of 40 mRNAs associated with Dectin-1 signaling and trained innate immunity [14]. We conducted the present exploratory investigation to identify additional mRNA targets of BYBG by broadly examining targets associated with immune cell maturation.

**Figure 1:**
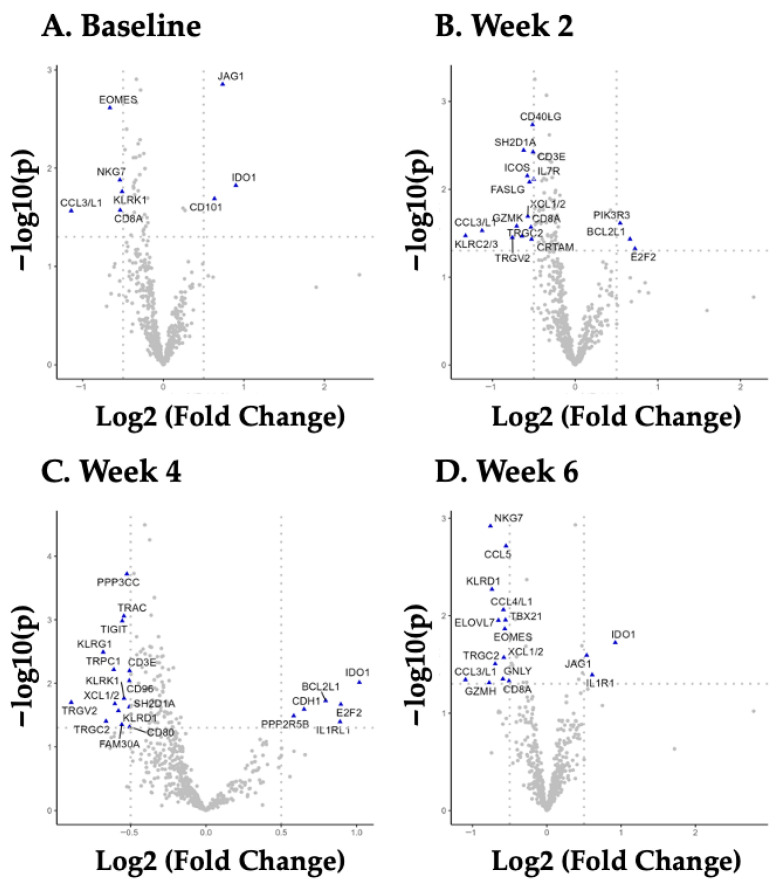
Change in mRNA with BYBG. Volcano plots reflect mRNA whose expression was significantly different from BYBG compared to the placebo. Since the baseline samples were collected prior to the start of the BYBG supplementation, the mRNA changed at this point, reflecting those that were different between participant groups prior to supplementation. The 42 mRNAs significantly (blue triangles) changed by oral BYBG supplementation were found using a multiplex mRNA array (NanoString).

In the present study, initial statistical testing identified 97 mRNAs that were significantly modulated with BYBG at one or more of the three time points (2, 4, and/or 6); however, after FDR control, only 42 mRNAs remained and were included in the final analysis as potential targets that were associated with BYBG supplementation. Figure 1 demonstrates the relative expression of the 42 mRNAs that were significantly changed by BYBG. The volcano plots in this figure demonstrate the mRNA changes relative to placebo, adjusted for individual baselines (pre-supplement response). When completing this comparison, it is important to note that eight mRNAs were significantly different between groups prior to supplementation (EOMES, NKG7, CCL3/L1, KLRK1, CD8A, JAG1, IDO1, and CD1010). It is possible that these pre-supplementation differences between groups may simply be a side effect of the small sample size in the present study, and future studies should be designed to fully evaluate these initial group differences. This means that 34 of the remaining mRNAs were uniquely changed at either weeks 2, 4, and/or 6. This potential diurnal change in mRNA expression with BYBG is very consistent with what we previously reported with BYBG and innate immune training mRNA [14].

The exploratory nature of the present study and its associated purpose prevented us from providing an in-depth discussion of the functional significance of the observed changes. Future studies that include both mRNA and traditional immune markers will be needed to complete that degree of functional comparison. Our objective in the present study was to identify potentially novel mRNA biomarkers of BYBG effectiveness without overextending the functional significance of what these changes may mean.

### 2.2. NanoString Annotated Pathways

Beyond the identification of individual mRNA, we also wanted to note existing immune response pathways that may be impacted by BYBG. To accomplish this secondary objective, we used annotated pathways provided by the manufacture of our analysis system (NanoString; Seattle, WA, USA). Since we used a standard, commercially available multiplex mRNA kit (Nanostring), the annotated pathways are well documented in the literature. The 21 NanoString annotated pathways that were changed by BYBG were as follows: antigen presentation, apoptosis, B cell memory, cell cycle, chemokine signaling, cytotoxicity, DAP12 signaling, hypoxia response, IL-1 signaling, IL-10 signaling, MAPK signaling, myeloid immune response, NF-kB signaling, NK activity, Notch Signaling, PD1 signaling, Senescence/Quiescence, T cell checkpoint signaling, TCR signaling, TLR signaling, and TNF signaling. These annotated pathways are associated with a variety of aspects of immune response that may be impacted by BYBG. Since most of the significantly changed mRNA expression (34 of 42) presented at 2, 4, and/or 6 weeks of BYBG supplementation, it seems reasonable to speculate that there may be a diurnal or duration of supplementation effect associated with BYBG. This duration of dosing effect is very consistent with what our lab and others have reported with respect to BYBG and changes in functional immune response [8,9,12,13,15,16,17,18]. Future research studies will be needed to fully understand the implications of the observed BYBG pathway changes on immune function and response.

### 2.3. Study Limitations

An a priori sample size analysis was conducted, which was consistent with the stated exploratory purpose of the present study, using our previous mRNA studies as a reference [12,14,23,24,25,26,27]. This analysis identified that a minimum of 16 individuals were needed to reach significance, and while we over-enrolled with 20 individuals, the sample size is still small. Given the small sample size and the large number of outcome measures, it is possible that the observed responses were not significant and potentially reflect a statistical error. We attempted to address this limitation by using a previously validated post hoc filtering method to reduce FDR. While methodologically justified and consistent with exploratory transcriptomic research, this approach may have introduced an element of subjectivity that may influence the interpretation. Although this approach helped mitigate the risk of spurious findings in the absence of multiple testing correction, it may also have increased the likelihood of excluding biologically meaningful mRNA that exhibited modest but potentially important changes. This is particularly relevant in immune-related gene expression, where small shifts in mRNA expression may have significant downstream effects on cellular function. The NanoString platform includes built-in controls and normalization strategies that reduce technical variability; however, FDR inflation was still possible. The population of individuals enrolled in the present study included healthy, young men and women who were interested in participating, representing a well-characterized, controlled population. Thus, while the observed findings are applicable only to our study population, these exploratory findings will support further study of BYBG in other, potentially immunocompromised populations.

### 2.4. Future Opportunities/Next Steps

The key objective of the present study was accomplished in the sense that we identified 42 new mRNA targets of BYBG supplementation. Our lab and others have used very similar models to detect changes in immune function with BYBG. While the present, exploratory study did not measure immune function, we did identify immune response annotated pathways that may be impacted by BYBG. For our next logical progression of this work, we will complete a larger study with a larger sample size to fully validate the relationship between BYBG-associated mRNA and immune function. While the present findings are hypothesis-generating, future validation may also focus on populations with immune deficiency or other lifestyle aspects that negatively impact immunity.

## 3. Materials and Methods

### 3.1. Experimental Approach and Ethical Considerations

This study adhered to the latest NIH Common Rule and received approval from the University of North Texas Institutional Review Board (IRB). Healthy adult participants with no diagnosed metabolic or inflammatory conditions provided both oral and written informed consent prior to enrollment. A total of twenty apparently healthy adults (age = 31 ± 4 years; biological female *n* = 10; BMI = 25.4 ± 1.2 kg/m^2^) were randomized into one of two groups: Baker’s yeast beta-glucan (BYBG; Wellmune^®^, Kerry Inc., Beloit, WI, USA) or placebo (maltodextrin). The BYBG used in this study was 100% beta-glucan with no fillers or excipients. An a priori sample size analysis conducted prior to the present study revealed that a minimum of eight individuals per treatment group was needed to significantly resolve mRNA that differed between the BYBG and the placebo (80% statistical power). The study was over-enrolled to ensure that post hoc changes in effect size did not negatively impact the outcomes. The final calculated statistical power for the present study exceeded 90%. Participants consumed 50 (weeks 0–2), 125 (weeks 2–4), and 250 (weeks 4–6) mg/d of BYBG during the 6-week study period by using a progressive dosing model that is similar to our previous study [25]. Maltodextrin has been used for many years as a placebo control when studying BYBG [13,14,18]. To assess the molecular effects of BYBG, differential mRNA expressions were evaluated relative to the placebo to identify transcripts that were uniquely altered by BYBG at weeks 0, 2, 4, and 6. Daily supplementation was administered via blister packs to ensure dosing accuracy, and participant compliance was 92%. The participants were scheduled to report to the laboratory for blood sample collection between 0600 and 1000 to minimize the diurnal effects. Also, a complete blood count was measured for each sample to ensure that the participants were fully hydrated. Further, the CBC revealed similar, not significant differences, in total leukocyte counts or the subset distribution over the course of all study visits.

### 3.2. Total RNA Extraction and mRNA Analysis

Total RNA was extracted and analyzed by using validated protocols that have been previously described by our laboratory [12,14,19,26]. Briefly, venous blood samples were collected into Paxgene RNA stabilization tubes (PreAnalytiX, Hombrechtikon, Switzerland) between 0600 h and 0900 h following an overnight fast (>8 h, water permitted) and at least 24 h of exercise abstention. RNA isolation was performed using an automated QIAcube system (Qiagen, Hilden, Germany) according to the manufacturer’s instructions. Gene expression profiling was conducted using a standard 784-plex Human Immune Exhaustion Panel on the NanoString nCounter platform (NanoString Technologies, Seattle, WA, USA). This panel was selected because it provided a comprehensive analysis of Immune Activation, Immune Suppression, Immune Status, Immune Checkpoints, Epigenetics, Metabolism, and Microenvironment, which may be altered with BYBG. Raw digital counts were acquired using the nCounter Sprint Profiler. Each assay included internal positive and negative controls, as well as a panel of housekeeping genes,= to ensure data quality and enable normalization.

### 3.3. Statistical Analysis, Quality Control, and False Discovery Rate

NanoString nCounter data were processed using the NanoTube R package (R v3.3.2) and Shiny application [20], which implements manufacturer-recommended normalization and quality control procedures. This application performs manufacturer-recommended normalization steps, including positive and housekeeping normalization, as well as the removal of target genes that have been found to have expression levels below ‘background’ (estimated from the negative control gene expression). These include positive control normalization, housekeeping gene normalization, and background thresholding using negative controls. Differential expression analysis was performed using the Limma package (the NanoTube R library also allows DE analysis using NanoStringDiff; (R v3.3.2)), integrated within NanoTube, to identify mRNA transcripts that were significantly altered by BYBG supplementation. The differential log_2_ fold change in gene expression was calculated separately for the BYBG and placebo groups by using baseline (week 0) correction for each group (i.e., BYBG vs. placebo). A comparative analysis identified mRNAs that were significantly upregulated or downregulated in response to BYBG. Statistical significance was defined as *p* < 0.05. In line with NanoString’s guidance, universal multiple comparisons correction was not applied so as to avoid inflating false negative rates. Instead, a post hoc strategy was used to balance Type I and Type II error risks, which is consistent with our prior discovery work [14]. This approach prioritized sensitivity while maintaining specificity, which is appropriate for the exploratory nature of this study.

The likelihood of false positives and false discovery rate (FDR) control was completed by excluding transcripts based on the following criteria: (1) The fold change in expression was between +0.5 and −0.5, (2) similar expression patterns were observed in both BYBG and placebo groups, and (3) there was no supporting evidence in the literature linking the mRNA to BYBG and immune modulation. This methodological approach was used instead of universal corrections since the goal of the present study was to identify targets for a larger study rather than to come to definitive conclusions.

## Data Availability

The raw data supporting the conclusions of this article will be made available by the authors on request.

## Abbreviations

The following abbreviations are used in this manuscript:

AKT3	AKT serine/threonine kinase 3
ATR	Ataxia Telangiectasia and Rad3 Related (ATR)
BCL2L1	BCL2-like 1
BYBG	Baker’s Yeast Beta Glucan
CCL3/L1	C-C Motif Chemokine Ligand 3 (also known as Macrophage Inflammatory Protein 1-Alpha
CCL4/L1	C-C Motif Chemokine Ligand 4 Like 1
CCL5	C-C Motif Chemokine Ligand 5
CD3E	CD3e Molecule, Epsilon
CD3G	CD3g Molecule, Gamma
CD40LG	CD40 Ligand
CD80	Cluster of Differentiation 80
CD8A	Cluster of Differentiation 8A
CD96	Cluster of Differentiation 96
CDH1	Cadherin 1, E-Cadherin (Epithelial)
CRTAM	Class I-Restricted T Cell-Associated Molecule
CXCR3	C-X-C Motif Chemokine Receptor 3
DAMP	Damage-Associated Molecular Pattern
E2F2	E2F Transcription Factor 2
ELOVL7	ELOVL Fatty Acid Elongase 7
EOMES	Eomesodermin
FAM30A	Family with Sequence Similarity 30 Member A
FASLG	Fas Ligand
FDR	False Discovery Rate
GNLY	Granulysin
GZMH	Granzyme H
GZMK	Granzyme K
ICOS	Inducible T Cell Costimulator
IDO1	Indoleamine 2,3-Dioxygenase 1
IL1R1	Interleukin 1 Receptor Type 1
IL1RL1	Interleukin 1 Receptor-Like 1
IL2RA	Interleukin 2 Receptor Alpha
IL7R	Interleukin 7 Receptor
JAG1	Jagged Canonical Notch Ligand 1
KLRC2/3	Killer Cell Lectin Like Receptor C2/C3
KLRD1	Killer Cell Lectin Like Receptor D1
KLRG1	Killer Cell Lectin Like Receptor G1
KLRK1	Killer Cell Lectin Like Receptor K1
LAG3	Lymphocyte Activating Gene 3
LPAR1	Lysophosphatidic Acid Receptor 1
NFKBIB	NF-kappa-B Inhibitor Beta
NKG7	Natural Killer Cell Granule Protein 7
PAMP	Pathogen-Associated Molecular Pattern
PIK3R3	Phosphoinositide-3-Kinase Regulatory Subunit 3
PPP2R5B	Protein Phosphatase 2 Regulatory Subunit B’ Beta
PPP3CC	Protein Phosphatase 3 Catalytic Subunit Gamma
RORA	RAR Related Orphan Receptor A
SH2D1A	SH2 Domain Containing 1A
TBX21	T-Box Transcription Factor 21
TIGIT	T Cell Immunoreceptor with Ig and ITIM Domains
TPP2	Tripeptidyl Peptidase II
TRAC	T Cell Receptor Alpha Constant
TRAT1	T Cell Receptor Associated Transmembrane Adaptor 1
TRGC2	T Cell Receptor Gamma Constant 2
TRGV2	T Cell Receptor Gamma Variable 2
TRPC1	Transient Receptor Potential Cation Channel Subfamily C Member 1
XCL1/2	X-C Motif Chemokine Ligand 1/2
